# Preventing and Investigating Horse-Related Human Injury and Fatality in Work and Non-Work Equestrian Environments: A Consideration of the Workplace Health and Safety Framework

**DOI:** 10.3390/ani6050033

**Published:** 2016-05-06

**Authors:** Meredith Chapman, Kirrilly Thompson

**Affiliations:** 1 The Appleton Institute, Central Queensland University, 44 Greenhill Road, Wayville, SA 5034, Australia; kirrilly.thompson@cqu.edu.au; 2 Safety in Focus, PO Box 711, Narrabri, New South Wales 2390, Australia

**Keywords:** horses, people, risk, mitigation, safety, WHS, injury, deaths, workplace

## Abstract

**Simple Summary:**

Attempts to reduce horse-related injuries and fatalities to humans have mostly focused on personal protective equipment like helmets. In organizational contexts, such technical interventions are considered secondary to reducing the frequency and severity of accidents. In this article, we describe the Workplace Health and Safety (WHS) framework that has been associated with reduced risks in industries and organisations. We consider how such a framework could be used to reduce horse-related risks in workplaces, as well as non-work equestrian competition and leisure environments. In this article, we propose that the simplicity and concepts of the WHS framework can provide risk mitigation benefits to both work and non-work equine identities.

**Abstract:**

It has been suggested that one in five riders will be injured due to a fall from a horse, resulting in severe head or torso injuries. Attempts to reduce injury have primarily focussed on low level risk controls, such as helmets. In comparison, risk mitigation in high risk workplaces and sports is directed at more effective and preventative controls like training, consultation, safe work procedures, fit for purpose equipment and regular Workplace Health and Safety (WHS) monitoring. However, there has been no systematic consideration of the risk-reduction benefits of applying a WHS framework to reducing horse-related risks in workplaces, let alone competition or leisure contexts. In this article, we discuss the different dimensions of risk during human–horse interaction: the risk itself, animal, human and environmental factors and their combinations thereof. We consider the potential of the WHS framework as a tool for reducing (a) situation-specific hazards, and (b) the risks inherent in and arising from human–horse interactions. Whilst most—if not all—horses are unpredictable, the majority of horse-related injuries should be treated as preventable. The article concludes with a practical application of WHS to prevent horse-related injury by discussing effective evidence-based guidelines and regulatory monitoring for equestrian sectors. It suggests that the WHS framework has significant potential not only to reduce the occurrence and likelihood of horse-related human accident and injury, but to enable systematic accident analysis and investigation of horse-related adverse events.

## 1. Introduction

Despite workhorses being largely replaced by machinery in the industrial era, horses have continued to ‘work’ for humans. They are our athletes, entertainers and therapists. Horses aid humans in law enforcement and agriculture, and take centre stage in racing. They are supported by an equine industry serviced by human specialists: riders, handlers, trainers, coaches, farriers, veterinarians, transporters and more. These humans are exposed to horse-related risks on a regular —if not daily—basis. Although the risks to humans of interacting with horses are well known [1,2,3,4,5], there have been no significant reductions in rates of injury or death over the past decades [6]. In contrast, there have been significant decreases in injuries and fatalities in high risk workplace settings such as mining and construction [7]. One significant difference between injury and fatality rates in high-risk industry compared to high-risk interspecies interactions across the combined sport and leisure sectors is Workplace Health and Safety (WHS). There has been widespread adoption and implementation of WHS principles in industry [8], with a focus on improved risk management. Broadly speaking, the same cannot be said for Australia’s horse industry where formal WHS application is inconsistent and restricted to select sectors like Thoroughbred Racing. This may result from diversity; with the equestrian sector including racing, sport, competition, recreation and leisure. Despite these activities making a significant economic contribution to Australia [9], they are not immediately—or equally—recognisable as workplaces. However, it seems prudent to consider if the application of a WHS framework to horse-related interactions across the broad horse industry could provide the same benefits in injury prevention and reduction that have been documented in other industries.

The aim of this article is to consider the potential benefits of applying a WHS framework to horse-related interactions to (a) reduce horse-related risks, and (b) enable investigation of adverse horse-related incidents. To determine the applicability of WHS to horse-related risks, we first discuss the multiple dimensions of risk in human–horse interactions: horse, human, environment and combinations thereof. Given its track record of reducing risks in workplaces, we consider how applicable WHS is to human–horse interactions in work and non-work contexts. We determine that the WHS framework can be easily translated to horse-related interactions. In fact, despite the WHS framework being developed without explicit reference to animals, we identify particular potential for WHS to address the inherent unpredictability commonly attributed to horses. Moreover, we identify an additional role for the WHS framework in guiding horse-related accident and injury investigations. To our knowledge, this is the first considered proposition for the usefulness of a WHS framework in reducing horse-related human injury and fatality. This article suggests a research agenda that outlines the empirical research necessary to evaluate our propositions and concludes with the challenge that other high risk industries have reduced workplace injuries and deaths with WHS, so what’s preventing equine from achieving similar results.

## 2. Horse-Related Risks

### 2.1. The Horse

The horse is considered an animal with a fight/flight instinct that humans spend many hours trying to train, coerce and desensitize to any adverse external stimuli [10,11]. Horses intrinsically are herd animals, with ‘leader’ and ‘follower’ instincts, having a mind of their own, which leads to ‘unpredictable’ behaviours [12,13,14], although predictability is largely dependent on human–horse knowledge and capabilities [5]. When placed in a situation where they feel threatened or insecure, horses can display dangerous behaviours of running, biting, crushing or kicking [15]. Research has determined that horses have a ‘fear memory’ that can be ‘turned on’ by human interaction, or may be ‘toned down’ (directed by a human) to enable the development of ‘trust’ [16,17,18]. Whilst there is more to learn and understand about horses as a species that directly affects safe and successful human–horse interactions [19], the majority of horse behavioural ‘problems’ are thought to be caused by equine confusion or a lack of understanding between the human and horse [20,21].

### 2.2. The Human

The degree of ‘risk’ that extends to horse riders or handlers can be affected by their own subjective perception of their capability and ‘horsemanship’ skill. High perceptions of ‘self-efficacy’ [22,23] could increase the likelihood of an injury or even death. A human’s level of horse experience and knowledge can be defined by their age, the number of horse interactive hours, along with their type of supervision and training [24,25]. When there is a deficit in one of these components, the likelihood of harm increases [24,26,27,28]. A particularly important determinant of horse-related risk is the suitability of horse and human combinations [29,30]. A mismatch at any stage during contact between the human and horse could potentiate a negative incident. Moreover, competitive drives amongst professional or ambitious equestrians can compromise their safety, in the form of ‘goal seduction’ [31,32].

Similarly, humans tend to devalue the importance of equine safety at point of sale [33], possibly where sellers can be seduced by financial return. For example, human desire for financial benefit might result in knowledge of undesirable horse traits and/or dangerous behaviours being withheld from a buyer. Alternatively, it may result from a buyer’s desire to own a horse regardless of such concerns (perhaps due to high self-efficacy in addressing them). More naively, selling unsuitable horses to riders may be facilitated by a lack of buyer expectation and devaluation of safety.

### 2.3. The Environment

A key element in human–horse interaction may be the environment in which the connection occurs. The environment may include the physical location and terrain, whether the area is confined by barriers (fencing, yard or crush), weather conditions and the degree of visibility for the horse, rider or handler [34]. Any one of these occurrences may affect consequence. If there is a distraction, other animals, unfamiliar or loud noises or a change in routine, the environment is changed from its initial state. Horses are very visual and reactive to changes in the environment as well as changes in the appearance of other humans and animals.

The environment can be considered through the concept of an ‘affective atmosphere’ [35]. A poor safety culture or environment in a workplace, can be described as untidy, having unfinished jobs and where workers take risks or short cuts, with little or no communication, [36]. A positive safety culture evolves from the combination of both individual and group efforts towards values, attitudes, goals and proficiency of an organisation’s WHS program [37,38,39,40]. The type of safety culture that exists at a workplace is determined by a broad, organisation-wide approach to safety management. A safety conscious manager empowers workers to prioritise safety, which translates into a safer work environment. Therefore, the feelings and behaviours of humans can determine the type of environment that is displayed and generated [41].

## 3. What is Workplace Health and Safety? WHS

All humans should have the right to be safe, maintain good health and enjoy life. This right extends to a workplace setting, with a belief that a worker can go to a work, perform their assigned duties and return home safely, injury free. This ideology will only be successful at work if all stakeholders (workers, employers, suppliers *etc*.) are thinking ‘safety first’, planning for unforeseeable events that cause harm and implement systems to manage harmful exposures. At its simplest, WHS is a set of ‘processes and standards’, mandated by legal obligations, that workers are expected to follow to promote and maintain their personal health, safety and welfare and that of others. WHS legislation defines the context of work for a person who conducts business or undertaking, whilst providing stakeholders with a clear understanding about their obligations (duty of care) and the consequences for neglecting them [42]. Furthermore, WHS legislation prescribes the need for those in control of a workplace to provide safe premises, safe machinery/materials, suitable training, supervision, work environment and facilities, supported by safe systems of work [43,44].

Many countries have adopted a regulatory WHS framework to assist workplaces in meeting their obligations and keeping workers healthy and safe. If safe work systems are adopted that are easily understood, workers and their families have more financial security, unencumbered by injury or death. By using a predetermined and industry specific WHS framework, employers benefit from uninjured workers through improved productivity and lost time at work. Some of the items in a WHS framework include the provision of defined policies, procedures and clear processes for worker communication and participation about their own work practices and safety. Providing workers with the opportunity for training and skills development, ensuring proactive risk management practices are implemented and monitoring, measuring and reviewing workplace activities. Items within the WHS framework require mandatory compliance. However, each workplace has the opportunity to develop and adopt their own set of processes to demonstrate ‘due diligence’ and compliance as reasonably practicable [45,46,47,48,49].

### Horses as ‘Working Animals’: Working Horse Safety

In Australia, the majority of notifiable deaths and serious injuries each year occur in what Australia’s WHS regulator classifies as the top five ‘high risk’ workplaces: Transport, Agriculture, Construction, Mining and Manufacturing. All of these industries have a legislated ‘duty of care’ with designated accountabilities and responsibilities for WHS, including mandatory compliance using a suitable risk management approach. In 2014, Australia reported 20 work-related traumatic injury fatalities due to falls from a height, with Agriculture being rated the second highest contributing industry with ‘horse related’ human deaths accounting for three (SWA, [50]). Many other potential accidents are prevented, due to suitable training in the use and maintenance of equipment, farm plant and machinery. Likewise, ‘working horses’ require maintenance of their hooves (‘tyres’), general health and nutrition (‘fuel’), with knowledge of their level of education and training (‘fit for purpose use’). However, horses are not explicitly classified as a ‘tool of trade’ when used in Agriculture. It would appear that the ‘risk’ of human injury resulting from horses at work, the importance of record keeping and information seeking about this ‘working animal’, is given less attention when compared to other high risk workplaces that reply on suitable and safe work equipment. Recognising this oversight provides a significant opportunity to improve how horse-related risks are managed in work and non-work situations. In particular, there is a need to explicate how horses and horse-related injuries ‘fit’ within industry and understand the legal obligations that result from any classification.

In general, prosecutions for any offence against WHS legislation have identified that a ‘duty of care’ is owed to a person due to the nature of the worker and employer relationship. Examples of human and horse work arrangements include cowgirls/jillaroos and cowboys/jackaroos working on cattle stations and feedlots, as well as stable hands, polo grooms, track work riders, animal transporters, coaches and instructors. When engaging a ‘worker’ either paid or unpaid, WHS law usually prescribes compliance with a set of risk management responsibilities, such as suitable training, supervision, consultation, monitoring and the implementation of hazard controls. In the case of human–horse interactions, all horses can be considered as workers, attended to by small to large hosts of human workers.

To date, attempts to reduce injuries amongst this human workforce have focussed on technical interventions such as back protectors, inflatable vests and frangible pins that reduce the risk of rotational falls at fixed obstacles [26,27,51,52]. However, technical intervention is not considered the most effective means of reducing injury [53]. Wearing a helmet is the ‘lowest’ or ‘least’ effective form of risk control within the WHS ‘Hierarchy of Controls’ [54,55,56,57]. Moreover, helmets generally only apply to riders (not handlers), who may only wear them when they are compulsory, such as during competition [58].

Many researchers have identified a need for attention to shift from a preoccupation with incident data to risk management and injury prevention [59,60,61,62]. More information is needed to identify the variances in human ‘risk perception’ [63] and the ‘beliefs’ that shape human behaviour and the environment in a variety of equine activities. This has commenced with preliminary studies on risk perception and the socio-cultural dimensions of risk amongst equestrians [5,58,64]. However, there has been no evaluation of the potential benefits of applying WHS principles to prevent and reduce horse-related injury and fatality. This is surprising, given that coronial findings and case law have identified the exact kinds of horse-related risk contributors that a generic WHS framework could easily prevent; an inability to recognise the level of risk; an unsuitable match of horse, rider or handler; and a lack of appropriate levels of supervision and training [65,66,67]. Whilst the ability for a WHS framework to introduce regulatory compliance is clear, how else might it systematically reduce horse-related risks? In the following sections, we consider how a WHS framework can be applied to human and horse interactions. We note its simplicity of documentation; procedures and reporting; risk assessment; skills assessment; training and supervision; and structured communication.

## 4. Applying the WHS Framework to Horse-Related Human Injury and Fatality

### 4.1. Documentation, Procedures and Reporting

To assist in reducing workplace injuries, standardization of safety processes is necessary. Standardization promotes organizational consistency and details requirements for best practice performance, whereby it then becomes the ‘backbone’ to continuous systems improvement. Moreover, a standardized WHS system assists an organization or industry to identify what information needs recording and monitoring to reduce human exposure to potential hazards. Clear, concise documentation, procedures and reporting processes support humans to rationalize critical safety decisions. A WHS system also provides ‘evidence’ for those making safety judgments in the event of an incident, whereby demonstrating ‘due diligence’ [68,69].

For human–horse interactions, ‘due diligence’ can be achieved by implementing inspection checklists of rider/handler equipment; noting and reporting environmental conditions; formulating safe work procedures; assessing equine level of ‘risk’; determining rider/handler capabilities and maintaining training records. All of these processes provide consistency and communicate any changes that may occur [70,71]. Having a structure for equine workers to follow promotes group cohesiveness. Furthermore, it identifies who is responsible and accountable for what; enabling a reporting mechanism for near miss events, injuries, hazards and highlights areas for WHS systems improvement [72]. In directing and documenting delegated worker duties and activities, a WHS system evolves, forming a framework for the production of safer human–horse relations.

### 4.2. Risk Assessment

In a generic WHS framework, risk assessment refers to identifying hazards such as equipment or plant. In the case of horse-related activities, horses are also a hazard, albeit one with significantly more autonomy and capable of exercising their own will in unpredictable ways [73]. The Victorian Injury Surveillance Unit at Monash University in Australia classified horses for the purpose of data collection as being a form of farm transport [74], Also, some WHS prosecutions suggest horses may ‘fit’ within the workplace during human–horse related undertakings.

From this perspective, proactive equine Risk Management (RM) [75] is about identifying, assessing and managing relevant risks prior to and during human and horse contact. A horse assessment may capture dangerous behaviours elicited during an exposure to various stimuli and situational circumstances, with the assessment occurring during a variety of conditions [76]. Earlier studies with similar assessment criteria are those measuring a horses response to ‘human approach’ [77], testing ‘social separation’ responses in a horse [78,79,80] and ‘bridge testing’ when a horse and handler cross over a novel surface [81,82,83]. Other scientific horse studies have measured physiological heart rate (HR) responses to various stimuli and challenging situations [84,85], however these are not easily measured and present some ambiguity given an increase in HR generally links to an increase in physical activity. A more complex horse assessment similar to a ‘novel object test’ [78,82,86] with a combination of assessments is needed for consistency.

Risk assessment should commence when a human first approaches and makes contact with a horse. The aim is to identify what positive and negative behaviours a horse displays during ground work activities such as catching, tying up, picking up feet, lunging, and during preparation for riding. To identify the suitability of a horse, an experienced handler/rider may perform a ‘test ride’ [87,88]. This allows for the observation and anticipation of undesirable behaviours and changes in the horse’s response whilst exposed to a variety of stimuli and obstacles. The degree of change in the horse’s response can give a subjective indication as to its level of sensitivity or de-sensitivity. Measuring a horse’s response, whilst it is in contact with various obstacles and activities could give the rider an indication of how risky this horse may be during future human contact, and for what kind of rider it is most suitable. This process provides an RM platform, to identify a risk rating for a horse e.g., low, medium, high or unacceptable, thus providing an indication of how risky this horse may be [89]. Based on these practical horse assessment guidelines, WHS may provide ‘reasonably practicable’ insight into a foreseeable or unplanned event during human and horse interaction, whereby limiting harmful exposures that may not otherwise be accounted for. However, further scientific research is needed to identify validated and reliable tools that can be used in the field along with an audit of pre-existing tools.

### 4.3. Skills Assessment

Skill matching to assigned activities can save lives, but in equestrian environments, making appropriate decisions about horse/rider combinations requires more than just an assessment of the horse. A similar assessment of the level of skill and ‘horsemanship’ of riders and handlers should also be undertaken [87]. This type of assessment would be based on determining a human’s level of competency in performing a required task or activity. This process is used in other workplaces where assessing a worker’s ability to perform the inherent demands of a job promotes a suitable match [90,91]. Furthermore, it identifies a workers capability to perform their duties safety, to determine if they require further education, training, supervision or a change in job task [41]. In equine workplaces, a handler/rider could demonstrate their practical skills in handling and riding a horse in a controlled environment.

Similarly to a horse assessment, the human would show their level of skill in approaching a horse safely, tacking-up a horse in preparation for riding, riding the horse through a series of activities and obstacles in various environments, before ceasing their interaction with the horse [92]. A set of parameters defining beginner skill level to an advanced rider would be tabled using similar activities to support more reliable outcomes. The assessor can determine a handler/rider level of competency when they either start to show unsafe horsemanship and the assessment is ceased or they reach a pre-determined level successfully without signs of incompetence.

### 4.4. Training and Supervision

The WHS framework supports competency based training regimes as the most effective method to produce safe outcomes in work performance [93,94,95,96]. It is only through training, supervision and experience that humans learn the skills necessary to make safe decisions, whereby protecting them from unnecessary work and non-work related injuries and deaths. Sharing experiences and relevant information maintains open channels for workers and others to address issues as they arise. Through demonstration of job activities, assessment and adequate supervision, a worker can improve their level of skill which results in better performance and reduced risk taking behaviors [97]. Training regimes approved by accredited training organizations such as Australian Skills Quality Authority (ASQA) are rarely disputed. Due to regulatory monitoring of training delivery and certification requirements, there is some level of reassurance that when a worker obtains a license, skill or qualification there is both visual and documented evidence that this is so.

In contrast, equestrian activities have a long history and are subject to many different philosophies, applications and cultures [98,99,100,101,102,103], as well as different types of horses with their own dispositions [29]. There is no single, agreed upon approach to interacting with horses, let alone a shared understanding of the ‘safest’ ways to interact. Indeed, it is easier to talk about equestrian sub-cultures and their conflict—such as classical equitation [104,105,106], modern equitation [29,64,107,108,109,110,111,112,113,114,115,116,117], natural equitation [99,101,102,118] and scientific equitation [20,119,120,121,122]—rather than an unchallenged equestrian culture. Current practices differ, are resistant to change, are antiquated or may have become dislocated from their original conditions. Some serve no other purpose than aesthetic pleasure, such as high school dressage, the purpose of which is frequently justified in relation to obsolete practices of military riding and clearing the ground of foot soldiers. Whilst the burgeoning field of equitation science is making significant progress in providing an evidence base to understanding equine ethology and identifying its implications for safe human–horse interaction, the existing WHS framework could be readily implemented to ensure that horse-related training and supervision is established as commonplace and subject to regular review.

To ensure effective training programs for safe horse handling skills, an individual training plan [123] needs to be developed, especially with workers engaging horses. This would include recognition of demonstrated prior knowledge and skill, forming a current assessment of handler/rider ability, and developing a plan to increase skills to a more advanced and unsupervised level. The process of learning would commence with a foundation in safe approach and handling of a horse (demonstrated on horses with a variety of handling experience, in confined and open spaces, over a designated duration), and discussion to identify underpinning knowledge relative to various horse-related activities. This would be followed by a rider validating their perceived level of riding skill in a controlled environment, progressing as deemed safe and competent to higher levels of demonstrated rider ability involving precision, control and completion of assigned activities [124]. Where a rider exhibits unsafe and/or inadequate skill, further training and supervision would be required to promote a riders ability to achieve an assigned task. For example, in the job of mustering cattle, if the skill level of a rider was rated at a lower level, they would ‘tail a herd of cattle’ where they are positioned riding at the rear and under supervision. As the riders’ skills increased they could ‘turn back cattle’ (retrieve those that escaped the herd) and eventually, as an advanced rider, ‘lead the herd of cattle’, being positioned up front. In any workplace where a duty of care exists between an employer and its workers (paid or unpaid), WHS legislation applies. The equine industry is no exception. The duty of keeping workers ‘healthy and safe’ (as reasonably practical) is clearly tabled by defined statutory duties (accountabilities and responsibilities) embedded within the WHS Acts and Regulations [125,126,127]. Where this mandatory obligation applies, every horse and human activity deemed as a work undertaking or workplace would benefit from the legal defense of using a WHS framework with a documented safety management system.

### 4.5. Structured Communication

Consultation and communication is a legal requirement and an essential part of managing safety risks. In order to achieve a safe workplace, everyone involved needs to communicate with each other to identify hazards and risks, talk about safety concerns and work together to find solutions [128,129]. Communication assists an organisation by drawing on the knowledge and experience of its workers, enabling more informed decisions to be made about how work should be carried out safety [130]. For communication to be successful, it needs to be easily understood, relevant and ‘effective’.

Developing ‘safe’ human–horse relationships requires the views of others being heard and greater co-operation and trust. Communicating important safety information such as poor horse behaviours, risks when handling or riding horses, rider capabilities, and/or emergency stop preparedness, prior to and during horse and human interaction will promote the delivery of a ‘best practice’ model. It provides feedback to support value added training programs, enhances safety awareness and promotes a ‘safety first’ culture. For example, making time to discuss horse health gives an opportunity to understand adverse horse reactions to pain or discomfort [131]. Prior to any horse and human interaction, communication may be relayed by a daily meeting, to generate discussions on pre-existing horse conditions, horse performance or health care needs (teeth, farrier, worming) and future management, similar to the patient handovers undertaken in hospital and care settings [132,133,134].

Communication can take the form of verbal informal chats; record keeping of horse health and treatments; evaluating a horse’s response to different activities; group discussions about exposures and handler/rider activities. Noting or reporting any subtle changes in a horse’s usual behaviour and herd responses are keys to managing safe horse and human interactions where this dynamic relationship is ever changing.

## 5. What Can We Do about What is Missing in the Equine Industry?

Every human has a ‘duty of care’ to one another, be that directive under Common, Civil or WHS law. The obligation is to demonstrate ‘due diligence’ and work to improve horse-related human injury or death by identifying suitable and plausible risk mitigation strategies. In Australia, ASQA has audited all horse related courses delivered nationally to ensure course competencies and training providers are maintaining ‘due diligence’ [135] (unpublished work). This is one step in the right direction, but more can be done to deliver better and safer equine safety and training standards, especially in those human–horse interactions that are not delivered by an ASQA accredited Registered Training Provider (RTO).

This article proposes that a WHS framework has significant potential to reduce horse-related injury and fatality in work as well as non-work contexts. One novel proof of concept is to use the WHS framework to structure a systematic review of horse-related human deaths where there are multiple sources of information that make it possible to: (a) reconstruct the event; (b) conduct a root cause analysis [136,137]; (c) identify key points at which a WHS framework was absent; (d) retrospectively reconstruct the event with the recommended WHS framework in place; (e) compare the hypothetical outcome with the actual event; and (f) identify improvements to prevent future incidents. By using investigative tools like root cause analysis and pre-event incident exploration, it should be possible to identify predisposing factors to the incident and theoretically consider what preventative safety measures could have been adopted, and should be routinely maintained. Having access to coroners’ reports, interviewing consenting injured parties and analyzing accident information against a safety standard (e.g., AS/NZ: 4801, ISO1400 or WHS Legislation) will provide valuable information about imminent ‘risks’ during horse and human interaction.

Further empirical research is required to determine how translatable a WHS framework and its benefits are in reducing horse-related human injuries and deaths in non-work environments. Such research will be essential to identifying areas of necessary adaptation or extension, barriers for implementation and resistance from the target audience, and existing fora to promote safety awareness education and initiatives. There is also a particular need to identify best practice industry role models [58], as future practical initiatives may benefit from involving those already implementing WHS successfully in work environments (e.g., Thoroughbred Racing) with participants in the non-work equine sector (e.g., as Pony Clubs).

## 6. Conclusions

The above discussion suggests that a WHS framework can support a reduction in horse-related human injuries and deaths through documented procedures and reporting; risk assessment; skills assessment; training and supervision and improve safety culture in the equestrian industry. By being ‘risk aware’ of the inherent dangers with horses and taking conscious steps to reduce harm, horse handlers and riders can become insightful and responsible, therefore endorsing safe behaviors that stimulate a proactive equine safety culture [40,138]. As such, a WHS framework has significant potential to reduce the risks inherent in, and arising from horse and human interaction, regardless of whether they occur in work, private, public, amateur or professional contexts. It could also pave the way for improved education and behavior change strategies, especially to overcome any fatalistic acceptance that horse-related human injuries and deaths are inevitable [40].

Given that there is a pre-existing framework of WHS that has had demonstrated success in other work contexts, any form of complacency around horse-related human injury and fatality is inexcusable. But how do we anticipate and ever hope to reduce horse-related human injury and fatality when equine associated legal obligations are still un-prescribed, with no best practice guidance or enforcement of safety principles? Moreover the problem remains; where do horses fit into in current WHS law amongst specific definitions such as ‘plant’, ‘structures’ and ‘substances’. This omission and failure of a clear definition of a ‘working animal’ (e.g., horses) used at work or during human–horse interaction in a work setting leaves this high risk activity open to interpretation.

Therefore, there is a pressing need for the adoption of minimal and consistent standards for qualifications, training, supervision, consultation, monitoring and the implementation of hazard controls in accordance with a defined hierarchy. Given the evidence that WHS has assisted in the reduction of work-related injuries and deaths, it seems sensible to take advantage of an established framework to guard the lives and livelihoods of those working, playing and competing with horses.
